# A Simple and Novel Modification of the Nebulization Mask to Improve Nebulization in the Supine Position

**DOI:** 10.4274/TJAR.2022.221120

**Published:** 2023-08-18

**Authors:** Ashutosh Kaushal, Harish Kumar, Vaishali Waindeskar, Jai Prakash Sharma

**Affiliations:** 1 Department of Anaesthesiology, All India Institute of Medical Sciences, Bhopal, India

**Keywords:** Angle piece, catheter mount, nebulization, nebulization mask, supine

**Dear Editor,**

Nebulization therapy is commonly used for pre-hospital and in-hospital (operating theatre, intensive care unit and emergency area) patient care.^1^ For this purpose, a nebulisation mask device is widely used, which consists of a Hudson mask, a medicine cup and an oxygen tube (Figure 1A). It is necessary to pass oxygen flow from the bottom of the medicine cup so that oxygen can go through the medicine and the medicine can change from liquid to mist form.

Although the conventional nebulization mask is beneficial when the patient is in a sitting or semi-sitting position, it has some limitations while the patient is in a supine position like drug spillage as the medicine cup becomes parallel to the ground as well as improper mist formation as the flow of oxygen not passed through the medicine from the bottom of the medicine cap (Figure 1B). It is also very difficult and cumbersome to make the drug chamber straight by holding the medicine cup by hand while the patient is in the supine position.

To overcome these problems, we suggest a novel modification of the nebulization mask kit (Romsons Pvt Ltd, India) to improve the nebulization in the supine position. For this purpose, we need one catheter mount and one angle piece (Figure 1A). The Hudson mask is detached from the medicine cup, and the proximal part of the angle piece is attached to the distal part of the medicine cup. Then the distal part of the angle piece is attached to the proximal part of the catheter mount. Finally, the distal part of the catheter mount is attached to the Hudson mask to make the final modified version of the nebulization device (Figure 1C).

The advantages of such a design would be better mist formation and drug delivery, and the medicine cup can be kept vertical so that there will be no drug spillage due to the drug chamber tilting while nebulising patients in the supine position (Figure 1D). Patients like spine injury patients who are restricted to be nebulised in the supine position in the intensive care unit and operation theatre primarily benefit from this technique.

Although, there is the possibility of turbulent flow and drug condensation at angles due to the usage of an angle piece and catheter mount between the mask and medicine cup to straighten the medicine cup.^2^

We recommend this simplified yet useful version of the conventional nebulization mask device for the nebulization of patients in the supine position.

## Figures and Tables

**Figure 1:**
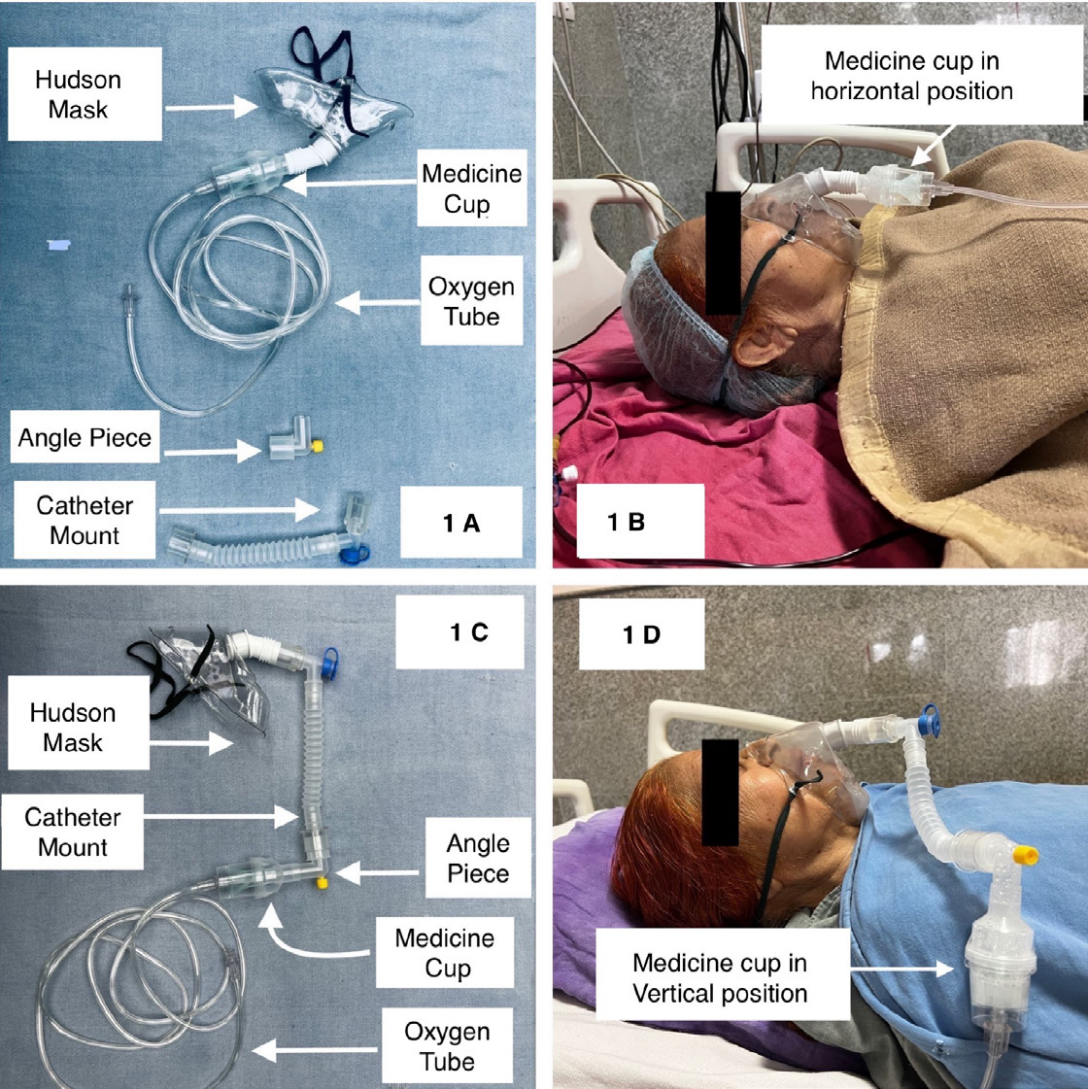
Modification of the nebulization mask.
